# Cost of wind energy generation should include energy storage allowance

**DOI:** 10.1038/s41598-020-59936-x

**Published:** 2020-02-19

**Authors:** Alberto Boretti, Stefania Castelletto

**Affiliations:** 1 Department of Mechanical Engineering, College of Engineering, Prince Mohammad Bin Fahd University, Al Khobar, Saudi Arabia; 2 School of Engineering, Royal Melbourne Institute of Technology (RMIT) University, Bundoora, Victoria Australia

**Keywords:** Batteries, Mechanical engineering

## Abstract

The statistic of wind energy in the US is presently based on annual average capacity factors, and construction cost (CAPEX). This approach suffers from one major downfall, as it does not include any parameter describing the variability of the wind energy generation. As a grid wind and solar only requires significant storage in terms of both power and energy to compensate for the variability of the resource, there is a need to account also for a parameter describing the variability of the power generation. While higher frequency data every minute or less is needed to design the storage, low-frequency monthly values are considered for different wind energy facilities. The annual capacity factors have an average of 0.34. They vary significantly from facility to facility, from a minimum of 0.15 to a maximum of 0.5. They also change year-by-year and are subjected to large month-by-month variability. It is concluded that a better estimation of performance and cost of wind energy facilities should include a parameter describing the variability, and an allowance for storage should be added to the cost. When high-frequency data will be eventually made available over a full year for all the wind and solar facilities connected to the same grid of given demand, then it will be possible to compute growth factors for wind and solar capacity, total power and energy of the storage, cost of the storage, and finally, attribute this cost to every facility inversely proportional to the annual mean capacity factor and directly proportional to the standard deviation about this value. The novelty of the present work is the recognition of the variability of wind power generation as a performance and cost parameter, and the proposal of a practical way to progress the design of the storage and its cost attribution to the generating facilities.

## Introduction

Wind energy facilities^1,2^ uses the variable wind energy resource to generate electricity. Wind energy is presently the most widespread and economic renewable energy^3^. While wind electricity supply is certainly less variable than solar photovoltaics, never available during nighttime and strongly affected by clouds and rain, but it still suffers from significant variations, over short as well as long time scales, because of the fluctuation of the wind energy resource^4,5^. The further uptake of the wind and solar photovoltaic components in a grid depends on the variability of the two sources, and the way to compensate for this variability, for example, trough energy storage^6^.

A proper study of wind energy supply needs high-frequency data of every wind energy facility connected to the same grid, as well as their sum. To match a given demand, supply from other resources must then compensate for the still intermittent and unpredictable total wind energy supply. In case of the further expanded capacity of wind energy also in excess of the grid demand, energy storage will have to accept the extra wind energy or supply the wind energy in defect of the demand. The costs of intermittency and unpredictability will have then to be shared between the individual wind energy facilities proportional to the variability of their supply. The availability of high-frequency data is therefore of paramount importance to study wind energy. Unfortunately, high-frequency generation data is very hard to be sourced worldwide for renewable energy facilities such as wind or solar. In the specific, high-frequency data is not available in the US, where the EIA only provide monthly average values.

A wind and solar only electricity grid requires a significant increment of the installed capacity of wind and solar, as well as the build-up of large storage, for both power and energy. There is a need to promote higher annual average capacity factors of wind energy facilities, and smaller fluctuations about these average values. The aim of this study is thus to assess real-world costs, annual average capacity factors, and parameters of the low-frequency fluctuations about these values, for the largest wind energy facilities of the continental US, excluding Alaska.

## The Present Assessment of the Cost and Performance of Wind Energy Facilities in the US

In the US^7^ reports the CAPEX (capital expenditure) plus the weighted average annual capacity factors of different Techno-Resource Group (TRG)^8–11^ areas, characterized by different average wind speed and wind speed range. There is no information about the variability of the capacity factors within the same TRG area.

Statistics for renewable energy including wind are often based on installed capacity (nominal power), rather than the actual power of generating energy across a year, see for example^7,12^ is under this aspect more accurate, providing the annual capacity factor, that coupled to the installed capacity, provides a measure of the actual electricity production over the year.

The US is one of the few countries where statistics are set-up in terms of energy rather than nominal power^13^ and^14^. Data on actual energy production is supplied for conventional and renewable energy plants in^15^, at low (up to monthly) frequency. High-frequency statistics would be necessary for proper assessments.

During 2017, within the US, wind turbines contributed 6.3% of the total utility-scale electricity generation, with remarkable growth from 6 to 254 billion kWh from 2000 to 2017^13,14^. While wind energy capacity (installed power) has increased dramatically over the last few years, wind energy electricity production has increased less. The average capacity factor has not increased, but reduced, with many wind energy facilities performing below expectations.

An introduction to wind energy in the US with the location of plants, completion date and number and size of turbines installed is provided in^16^. This assessment is not up to date.

On-shore based wind energy facilities in the US are discussed in^7^ as well as^17^, based on the works^18–22^ and^23^. These wind plants have a capacity in the range from 50 MW to 100 MW^19^, with an average 2-MW turbine having a rotor diameter of 102 m and hub heights of 82 m. These were the facilities installed up to 2015^20^.

Ref. ^21^ defines the renewable energy technical potential as the achievable energy generation per specific turbine installation. The technical potential is quantified by capacity factors. The capacity factors estimated were also based on five different wind turbines, optimized for the range of average annual wind speed at the locations. The chosen wind turbine power curves were representative of the range of wind plant installations in the US in 2015^20^. The capacity factor is referred to as an 80-m, above-ground-level, inter-annual average hourly wind resource data.

Most installed US wind plants align with ATB estimates for performance in Techno-Resource Groups (TRGs) 5–7. High wind resource sites (associated with TRGs 1 and 2) and exceptionally low wind resource sites associated with (TRGs 8–10) are not as common in the historical data. From^19^ and^23^, it may be expected for TRG1 a Wind Speed Range 8.2–13.5 m/s, average 8.7 m/s, and a 47.4% capacity factor, while for a TRG10 the Wind Speed Range is 1.0–5.3 m/s, average 4.0 m/s, for an 11.1% capacity factor. In the most common conditions of TRG 5 to 7, the wind speed range is 6.9–11.1 to 5.4–8.3 m/s, the average speed is 7.5 to 6.2 m/s, and the capacity factor is 40.7 to 30.8%^17^ and^7^ also supply future projections based on high, medium, and low-cost estimates based on the results of a survey of 163 of the world’s wind energy experts^21^.

Ref. ^7^ summarizes the forecasted CAPital EXpenditures (CAPEX) in $ per unit capacity (power, kW), and annual average capacity factor (ratio of energy produced in kWh to the product of capacity in kW by the number of hours in a year). In a location such as Los Vientos, TX, of prevailing wind speed about 7.5 m/s, we may consider the CAPEX values of TRG5, wind speed range 6.9–11.1 m/s, weighted average wind speed 7.5 m/s The weighted average CAPEX is 1,616 $/kW, and the weighted average capacity factor is 40.7%. The weighted average annual capacity factor of 2014 is 40–44%, and it is growing.

Unfortunately, high-frequency generation data are not available for the US, and proper energy storage computations are completely missing in the literature.

## Materials and Methods

Low frequency monthly average data of electricity production available from the US EIA is analyzed to show the variability of capacity factors month-by-month, year-by-year, and facility-by-facility, also in locations sharing about the same wind energy resource. A numerical method will then be used to explain the variability of the capacity factors in locations of about the same wind energy resource.

The method used here is from^6^. The wind energy facility nameplate capacity (power) is conventionally given as the sum of all turbine rated capacities (see^17^ and^7^). The instantaneous power of a turbine *P* depends on wind speed U, turbine swept area A and air density *ρ*, according to the equation:

1 $${P}_{i}=\eta \cdot \frac{1}{2}\cdot \rho \cdot A\cdot {U}^{3}$$

where *η* is the total turbine efficiency, including aerodynamic efficiency, the efficiency of power transmission, and the efficiency of electrical generation. Because of the Betz limit^24,25^ the aerodynamic efficiency cannot exceed 16/27 or 59.3%. Utility-scale wind turbines have peak aerodynamic efficiency of 75% to 80% of the Betz limit. Density *ϱ* and speed *U* vary in time and space, across the rotor area, and from one turbine to another. The rated capacity P_r_ is obtained at a reference, uniform speed *U*_*r*_ that is usually exceeding the most probable wind speed of a specific location, with a uniform reference density *ϱ*_*r*_.

From the net installed capacity *P*_*r*_, annual and monthly capacity factors *ε* are computed by dividing the annual and monthly electricity production *E*, downloaded from^15^ by the capacity *P*_*r*_ and the number *n* of hours in a year or a month:

2 $$\varepsilon =\frac{E}{{P}_{r}\cdot n}$$

Wind turbine power data are available from manufacturers. The wind turbine database of ^26^ includes data from 1759 different turbines from 424 manufacturers. The data are valid for reference density conditions and wind speed at hub height, in about optimal flow conditions, where the short-term average and turbulent values are uniform across the rotor area. Computer-Aided Engineering (CAE) tools such as those of ^27^, may also supply an estimation of the performances of a turbine of a given geometry. These tools are the industry standards for the design and analysis of wind turbines.

As actual operating conditions differ, the actual power production for a given prevailing wind speed may be different. Different hub heights are also possible for every turbine, translating into different wind speeds. The detailed information about wind speed and direction and air density, both short-term average and turbulent across the rotor area of every individual wind turbine of a wind energy facility installation are usually not available. The experimental data of wind resource is usually available only near the proposed wind energy facility installation, and not at hub height, but at ground level. This resource assessment is also limited to time windows that are not long enough to include climate variability on a multi-decadal scale^28,29^, and^30^.

The theoretical energy production for every month is obtained by integrating in time the power computed from the given wind speed at hub height. A correction factor is introduced to account for the monthly average temperature at ground level, which affects the air density at the rotor hub. The ground temperature is obtained by using the air temperature data of ^31^.

A simple model is defined to compute the electricity production of a wind turbine from the measured power curve. The wind speed at a reference hub height of 100 m is extrapolated from the speed at 10 m obtained by using the wind speed data of ^31^. In wind energy assessment, assuming a neutral atmosphere, two models are usually used to represent the vertical profile of wind speed over regions of homogenous, flat terrain. The first approach is the logarithm law. The second approach is the power law. Both approaches are subject to uncertainty. Mixing length theory, eddy viscosity theory, and similarity theory provide a logarithmic wind profile with height^32,33^. Alternatively, a wind profile power law may be used^34,35^. The exponent γ is an empirically derived coefficient that varies depending upon the stability of the atmosphere. Here we use a small exponent γ = 0.125 in the power-law returning the same velocity multiplying factor of 1.33 from 10 to 100 meters’ height, of a wind profile logarithm law with terrain roughness of 0.01 m. Data at 10 m, to be transformed in data at 100 m (rotor height) log law 1.33 multiplication factor. The density of air is 1.205 kg/m^3^ at 20 °C (293.15 K). Temperature reduces with altitude 0.98 °C per 100 meters.

It may be argued that the source and characteristics of the weather data are not representative of the conditions at the turbines, and that simply multiplying 10 m wind speeds by 1.33 is not really a valid way to find elevated wind speeds, as the atmospheric structure, and the orography, is much more complex than that the simple parameterization would imply.

The theoretical electricity production E is finally:

3 $$E=\beta {\int }_{{t}_{2}}^{{t}_{1}}P(\alpha \,U(t))dt$$

where *U* is the reference velocity measured at the time *t*, *α* is a correction coefficient to compute the velocity at hub height, *P* is the tabulated power function, and *β* is a correction factor for the density effect. *β is* taken as the ratio of reference temperature for the power curve, and average temperature over the time interval.

### Experimental capacity factors of wind energy facilities in the US

**Figure 1:**
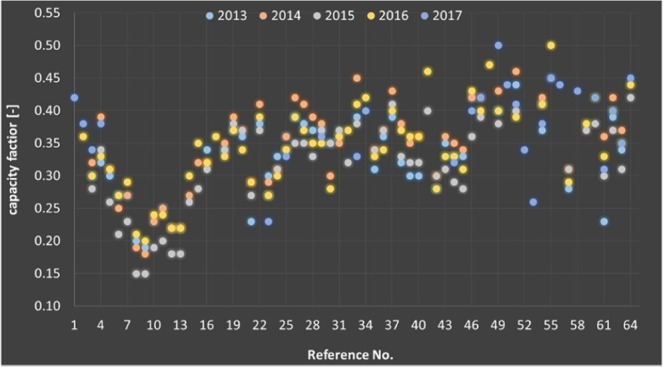
Capacity factors of wind energy facilities in the contiguous continental US. Data from^15^. Credit the U.S. Energy Information Administration (EIA).

An up-to-date description of the actual onshore wind energy facilities in the US is provided in Fig. 1. The 64 wind energy facilities considered are numbered 1 to 64, as shown in Table 1, that is reporting the name, capacity, capacity factor and type of turbines. Over the years 2013 to 2017, these wind energy facilities have run at capacity factors ranging from 15% to 50%, with an average of 34%. Data is from^15^. The most part of the wind energy facilities is in TRGs 5–7, where the Weighted Average Net capacity factors should range from 30.8% to 40.7%.

**Table 1:** Capacity and Capacity Factors of wind energy facilities in the contiguous continental US. Data from^15^. Credit the U.S. Energy Information Administration (EIA).

No	power plant	capacity factor					capacity [MW]	turbines
		2013	2014	2015	2016	2017		
1	Los Vientos IV	NA	NA	NA	NA	0.42	200	100 Vestas V110 2 MW
2	Los Vientos III	NA	NA	NA	0.36	0.38	200	100 Vestas V110 2 MW
3	Los Vientos 1B	0.30	0.32	0.28	0.30	0.34	202	84 Mitsubishi MWT 102 2.4 MW
4	Los Vientos 1 A	0.32	0.39	0.34	0.33	0.38	200	87 Siemens SWT 108 2.3 MW
5	Alta Wind Energy Center I	0.30	0.31	0.26	0.31	NA	150	100 GE SLE 1.5 MW
6	Alta Wind Energy Center II	0.27	0.25	0.21	0.27	NA	150	50 Vestas V90 3 MW
7	Alta Wind Energy Center III	0.27	0.27	0.23	0.29	NA	150	50 Vestas V90 3 MW
8	Alta Wind Energy Center IV	0.20	0.19	0.15	0.21	NA	102	34 Vestas V90 3 MW
9	Alta Wind Energy Center V	0.19	0.18	0.15	0.20	NA	168	56 vestas V90 3 MW
10	Mustang Hills (Alta Wind VI)	0.23	0.23	0.19	0.24	NA	150	50 Vestas V90 3 MW
11	Pinyon Pine I (Alta Wind VII)	0.25	0.25	0.20	0.24	NA	168	56 vestas V90 3 MW
12	Alta Wind VIII	0.22	0.22	0.18	0.22	NA	150	50 Vestas V90 3 MW
13	Pinyon Pine II (Alta Wind IX)	0.22	0.22	0.18	0.22	NA	132	44 Vestas V90 3 MW
14	Alta Wind X	NA	0.27	0.26	0.30	NA	138	46 Vestas V90 3 MW
15	Alta Wind XI	NA	0.32	0.28	0.35	NA	90	30 Vestas V90 3 MW
16	Adair Wind energy facility	0.34	0.32	0.31	0.32	NA	174.8	76 Siemens SWT-2.3-93
17	Wind IX Adams County	NA	NA	NA	0.36	NA	154.3	64 turbines 2.4 MW
18	Adams Wind energy facility	0.34	0.35	0.34	0.33	NA	19.8	12 Alstom 1.65 MW Ecotecnia 86 *
19	Adams Wind Generations LLC	0.37	0.39	0.38	0.37	NA	19.8	12 Alstom 1.65 MW Ecotecnia 86
20	Ainsworth Wind	0.36	0.37	0.37	0.34	NA	59.4	36 Vestas V82/1650
21	Allegheny Ridge Wind energy facility	0.23	0.29	0.27	0.29	NA	80	40 turbines 2 MW
22	Anacacho Wind energy facility, LLC	0.38	0.41	0.37	0.39	NA	100	NA
23	Biglow Canyon Wind energy facility	0.30	0.29	0.27	0.27	0.23	450	76 Vestas v82 1.65 MW + 141 siemens SWT 93 2.3 MW
24	Shiloh Wind Project 2 LLC	0.33	0.31	0.31	0.30	NA	150	75 REpower MM92 2 MW
25	Rolling Hills Wind energy facility	0.36	0.36	0.34	0.34	0.33	443.9	193 Siemens 2.3 MW
26	EC&R Panther Creek Wind energy facility I	0.39	0.42	0.35	0.39	NA	142.5	95 GE 77 1.5 MW
27	EC&R Panther Creek Wind energy facility II	0.38	0.41	0.35	0.37	NA	115.5	77 GE 77 1.5 MW
28	EC&R Panther Creek Wind energy facility III	0.37	0.39	0.33	0.35	NA	199.5	133 GE 77 1.5 MW
29	Pioneer Prairie Wind energy facility	0.37	0.38	0.37	0.35	0.36	300.3	182 Vestas V82 1.65 MW
30	Sherbino I Wind energy facility	0.35	0.30	0.35	0.28	NA	150	50 Vestas V90 3 MW
31	Whispering Willow Wind energy facility	0.36	0.35	0.37	0.36	NA	200	121 turbines 1.65 MW
32	Tucannon River Wind energy facility	NA	NA	0.32	0.37	NA	266.8	116 Siemens SWT 108 2.3 MW
33	Tatanka Wind Power LLC	0.39	0.45	0.38	0.41	0.33	180	NA
34	Balko Wind	NA	NA	NA	0.42	0.40	299.7	162 GE 87 1.85 MW
35	Bent Tree Wind energy facility	0.31	0.34	0.34	0.33	NA	201	NA
36	Bishop Hill 1	0.36	0.37	0.37	0.34	NA	200	NA
37	Bishop Hill II	0.39	0.43	0.41	0.40	NA	80	50 turbines: GE Energy 1.6–100
38	Bison Wind I	0.32	0.38	0.33	0.37	NA	82	16 Siemens SWT-2.3-101 + 17 Siemens SWT-3.0-101
39	Bison Wind II	0.30	0.35	0.32	0.36	NA	105	35 Siemens SWT-3.0-101
40	Bison Wind III	0.30	0.36	0.32	0.36	NA	105	35 Siemens SWT-3.0-101
41	Bison Wind IV	NA	NA	0.40	0.46	NA	205	64 Siemens SWT-3.2-113
42	Blackstone Wind energy facility II	0.28	0.30	0.30	0.28	NA	200	NA
43	Buffalo Gap Wind energy facility I	0.35	0.36	0.31	0.33	NA	120.6	67 Vestas V80 1.8 MW
44	Buffalo Gap Wind energy facility II	0.33	0.35	0.29	0.33	0.32	232.5	155 GE 1.5 MW
45	Buffalo Gap Wind energy facility III	0.33	0.34	0.28	0.31	NA	170.2	74 Siemens 2.3 MW
46	Canadian Hills Wind	0.42	0.42	0.36	0.43	0.40	298.45	62 Mitsubishi MWT 102 2.4 MW + 73 Senvion MM92 92 2.05 MW
47	Caney River Wind Project	0.42	0.42	0.39	0.40	0.42	201	NA
48	Cedar Bluff	NA	NA	NA	0.47	NA	199	NA
49	Chisholm View Wind	0.40	0.43	0.38	0.40	0.50	235	NA
50	Courtenay Wind energy facility	NA	NA	NA	NA	0.44	200	NA
51	Crossroads Wind energy facility	0.44	0.46	0.40	0.39	0.41	227	NA
52	Deerfield	NA	NA	NA	NA	0.34	149	NA
53	Desert Wind energy facility	NA	NA	NA	NA	0.26	208	NA
54	Fenton Wind energy facility	0.37	0.42	0.41	0.41	0.38	206	NA
55	Grandview	NA	NA	0.45	0.50	0.45	210.42	NA
56	Grande Prairie	NA	NA	NA	NA	0.44	400	200 Vestas V110 2 MW
57	Grand Ridge	0.28	0.31	0.31	0.29	NA	210	NA
58	Great Western Wind	NA	NA	NA	NA	0.43	225	NA
59	Headwaters Wind energy facility	NA	NA	0.37	0.38	NA	200	NA
60	Hereford 1	NA	NA	0.38	0.42	0.42	200	NA
61	Horse Hollow Wind Energy Center	0.23	0.36	0.30	0.33	0.31	736	291 GE 1.5 MW + 139 Siemens 2.3 MW
62	Limon Wind I	0.39	0.42	0.37	0.40	0.40	200	125 GE 100 1.6 MW
63	Limon Wind II	0.34	0.37	0.31	0.35	0.35	200	125 GE 100 1.6 MW
64	Limon Wind III	NA	NA	0.42	0.44	0.45	205.7	121 GE 100 1.7 MW
	min	0.19	0.18	0.15	0.20	0.23		
	max	0.44	0.46	0.45	0.50	0.50		
	average	0.33	0.34	0.32	0.34	0.38		

Figure 1 and Table 1 show significant differences vs. the pattern depicted in^7^, which appears optimistic. For example, for 2013^7^, reports weighted averages capacity factors of 35 to 39% and oscillations about these values from 26 to 49%. The naïve average of Table 1 for 2013 is 33%, with a minimum of 19% and a maximum of 44%. For 2014^7^, reports weighted averages capacity factors of 39% to 44% and oscillations about these values from 29 to 51%. The naïve average of Table 1 for 2014 is 34%, with a minimum of 18% and a maximum of 46%. Hence^7^, generally appears optimistic, overrating the average, minimum and maximum capacity factors.

The previous results have shown significant differences between the different wind energy facilities. These differences are due to a different resource, as well as a different design of the wind farm. Here we show as also wind energy facilities sharing about the same resource have different performances. The selected case study is Los Vientos, TX.

Ref. ^36^ presents the land-based wind speed at 100 m hub for the contiguous states of the US excluding Alaska with enlarged the area of south Texas where the Los Vientos wind energy facility complex is located. Los Vientos has 5 wind energy facility units in about the same location for wind resources. This is a good example to show the variability of the capacity factors from one unit to the other. Texas is one of the preferred areas for wind energy in the US, for both wind resources and topography. The area of Los Vientos has a prevailing wind speed approaching 7.5 m/s.

The complex has a total capacity of 910 MW. Unit I (or 1 A) and II (or 1B), of power 200 and 202 MW, began operation in December 2012. Unit III, of power 200 MW, started operations in May 2015. Unit IV, also of power 200 MW, came online in August 2016. Unit V, of power 110 MW, became operational in December 2015. Data is proposed by^37^.

Los Vientos I has 87 turbines Siemens SWT-2.3–101 of power 2,300 kW, diameter 101 m, and hub height 100 m. Latitude is 26°20′7.8″. Longitude is −97°38′51.4″. Los Vientos II has 87 turbines Mitsubishi MWT-102-2.4 of power 2,400 kW, diameter 102 m, and hub height 80 m. Latitude is 26°20′25.5″. Longitude is −97°41′19.6″. Los Vientos III has 100 turbines Vestas V110/2000, of power 2,000 kW, and diameter 110 m. The hub height is not known. Latitude is 26° 20′ 47.2″. Longitude is −97°36′58.6″. Los Vientos IV has 100 of the same turbines Vestas V110/2000, of unknown hub height. The precise localization is not given. Los Vientos V has 55 of the same turbines Vestas V110/2000, of unknown hub height Latitude is 26°37′17.4″. Longitude is −98°44′53.2″.

The experimental capacity factors for the Los Vientos units 1A, 1B, III and IV (there is no production data yet for unit V) are presented in^38^. The measured seasonal variability of capacity factors partially agrees with the reference seasonal variability for the lower plains region that is shown in^39^. This reference seasonal variability was based on data of facilities with a net capacity >1 MW and above within the specific area over the period 2001–2013. The seasonal and inter-annual variability of the capacity factors for this and other wind energy facilities are discussed in^38^.

During the year 2017, *ε*_*max*_, *ε*_*min*,_ and *ε*_*ave*_ were 48.58%, 20.84%, and 37.85%. This is slightly less than the 40.7% weighted average net capacity factor of a TRG5 location (wind speed range 6.9–11.1 m/s, weighted average wind speed 7.5 m/s)^7^.

Over the 5 years of data from 2013 to 2017, unit 1A had an average, maximum and minimum *ε* of 35.31%, 39.16%, and 32.49%, while unit 1B had an average, maximum and minimum *ε* of 30.93%, 33.76% and 28.02%. Unfortunately, there is not enough data for the other wind energy facilities to compute the inter-annual variability.

The difference vs. the reference seasonal variability for the lower plains of ^39^ is significant over every year.

Los Vientos 1 A outperformed Los Vientos 1B in terms of energy of 1.99% in 2013, 6.75% in 2014, 6.01% in 2015, 2.91% in 2016 and 4.07% in 2017^38^. Los Vientos 1A and Los Vientos III had about the same performances in 2017. Los Vientos IV outperformed both Los Vientos 1A and Los Vientos III in 2017. Orography and different relative locations of the turbines affect the result, same as turbine type and hub height.

**Figure 2:**
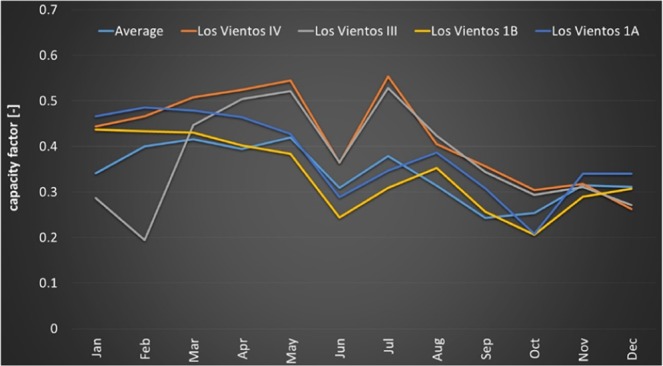
Monthly capacity factors during the year 2017 in Los Vientos. Image reproduced modified after^6,38^.

Figure 2 highlights the capacity factors of 2017 to better evidence the seasonal variation of this specific year. The Los Vientos III and IV wind energy facilities seem to work better during the middle months, while at the beginning of the year, the operation of Los Vientos III was far from optimal and at the end of the year, the advantages are lost. The Los Vientos 1A and 1B wind energy facilities have remarkably close performances despite the different turbines, with better performances of 1A the likely result of a higher hub height. Los Vientos IV had a 2017 capacity factor of 42.10%. Los Vientos III, of the same turbines, had a 2017 capacity factor of 37.56%. Los Vientos 1B, featuring different turbines, likely at a lower hub height than those of Los Vientos IV and III, had a 2017 capacity factor of 33.72%. Finally, Los Vientos 1A featuring other turbines had a 2017 capacity factor of 37.79%. Los Vientos 1B has capacity factors always smaller than Los Vientos 1A.

The monthly capacity factors of the Los Vientos wind energy facilities over the full length of their operation have been 10.61% to 55.32%, an average 33.91%. During 2017, the monthly capacity factors vary between 19% and 55%, with an average of 38%.

### Computational study of wind energy in Los Vientos, TX

After having shown that also wind energy facilities sharing the about the same resource have different performances, the effect of one of the design parameters, the power curve of the turbines, is here analyzed. It is common practice to take as the total installed capacity of a wind energy facility the sum of the rated powers of all the turbines. Other design parameters such as hub height, and relative position of every turbine in arrays, and influence of the orography, are typically neglected in computing the total installed capacity.

**Figure 3:**
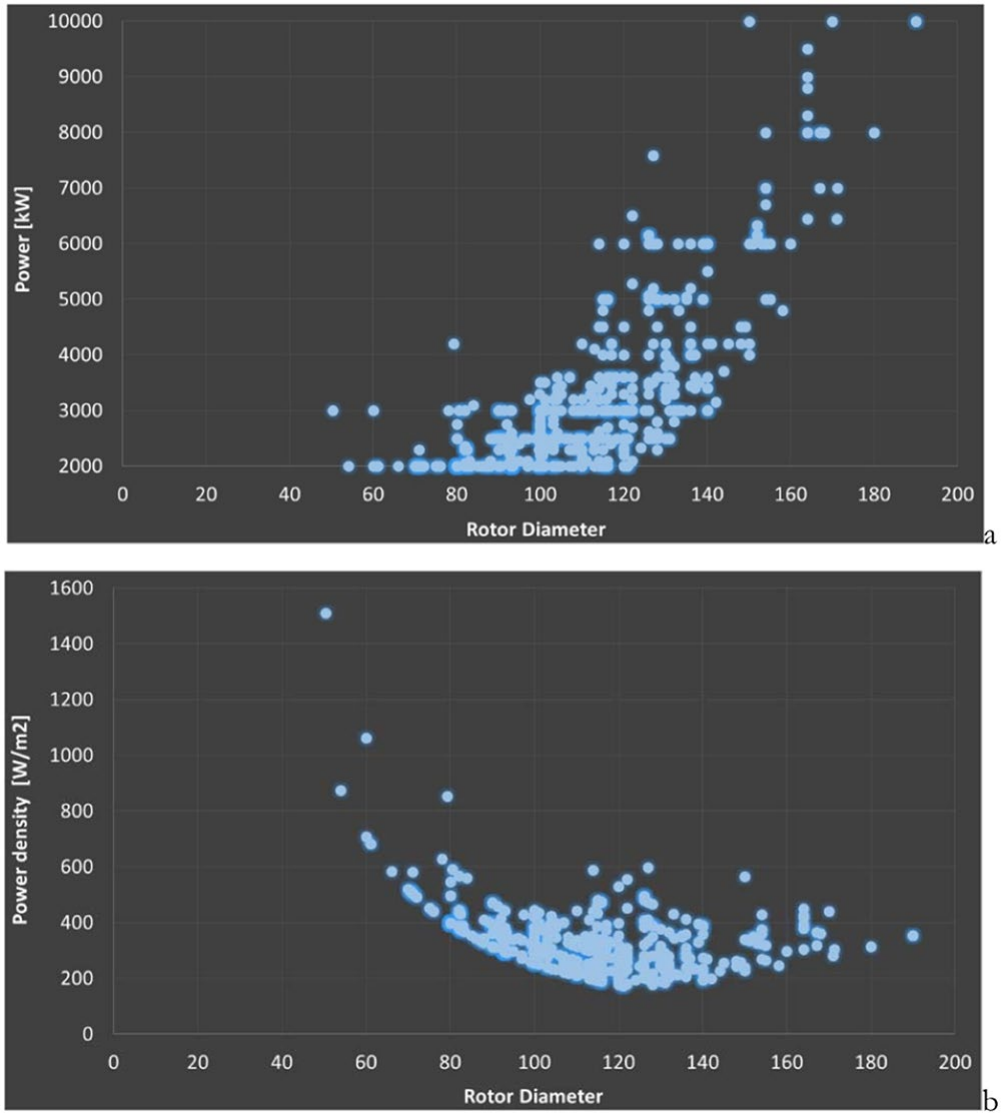
Power (**a)** and power density (**b**) of 553 turbines with power range 2 to 10 MW vs. rotor diameter.

Wind turbine data shows significant variability across designs. Figure 3 presents the data of power and power density, of 553 different turbines having power ranging from 2 to 10 MW as a function of the rotor diameter, the most relevant design parameter of a wind turbine. All the turbines are axial flow, 3 blades. The rated speed used to compute the power varies from one design to the other. This explains the spreading of the two distributions.

**Figure 4:**
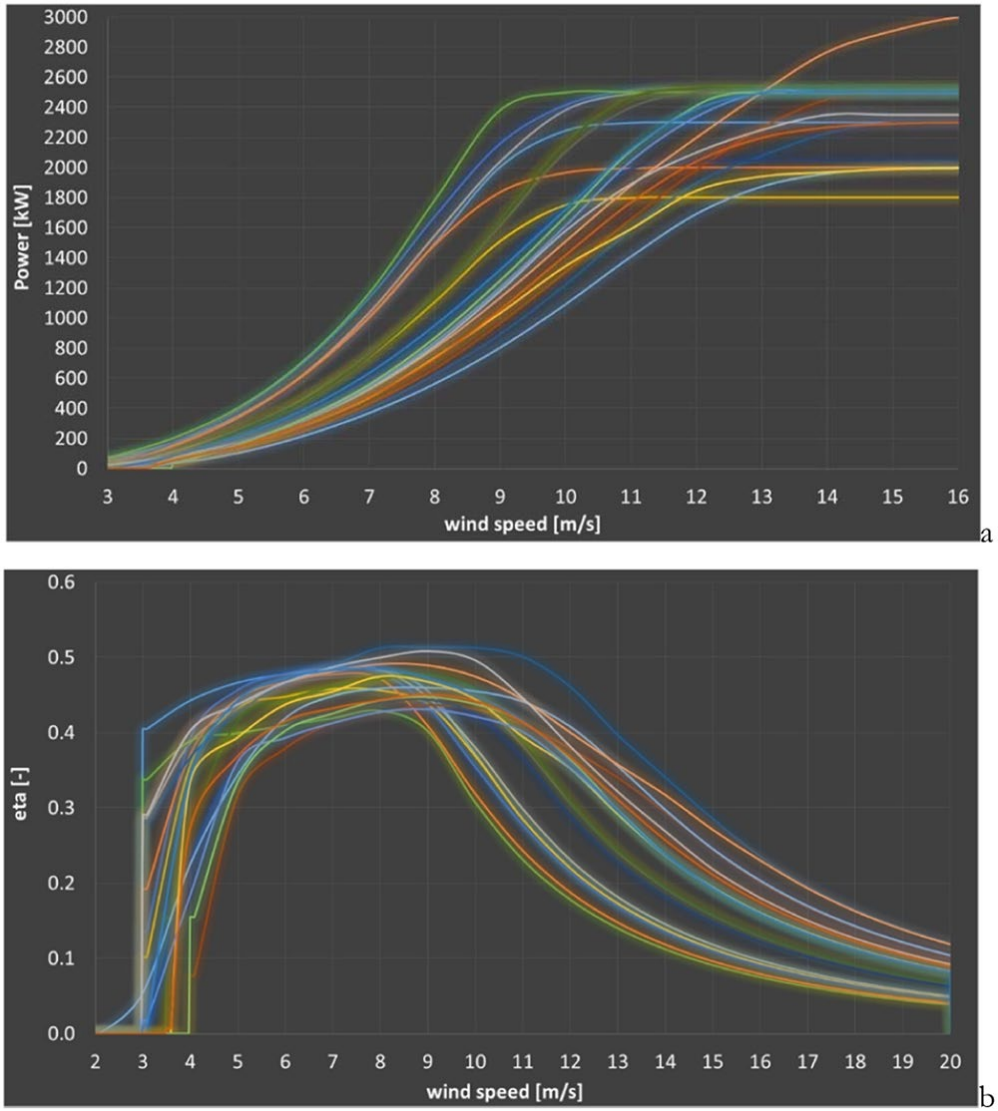
Power (**a**) and efficiency (**b**) curves of 20 turbines with a range of power 1.8 to 3.0 MW vs. wind speed. Images reproduced modified after^6^.

The top efficiency η is not reached at the rated speed, but well below. The most common operation of the turbines occurs at wind speeds below the rated speed. Modeling of wind turbines’ power curves, when not available from manufacturers, is discussed in^40,41^. Figure 4 presents the power and efficiency η curves of a subset 20 turbines of power 1.8 to 3.0 MW (data from^26^). The wind speed for η_max_ ranges from a maximum of 9 m/s to a minimum of 7 m/s with an average of 8.2 m/s. The η_max_ ranges from a maximum of 51% to a minimum of 43% with an average of 47%. The rated speed drastically changes from one design to the other. Higher power densities are found at higher rated power wind speed. Rated speed ranges from 10 to 16 m/s, with an average of 12.8 m/s. The figure shows the spreading of wind turbine power curves.

While the information available for the turbines of Los Vientos 1B, III and IV is insufficient to develop a model (the specific power curves are unavailable in^26^, and the hub height is similarly unspecified for III and IV), a simple model can be developed for Los Vientos 1A.

Los Vientos 1A has 87 turbines Siemens SWT-2.3-101 (power 2,300 kW, diameter 101 m)^26^. has no power curve for this turbine, having rated power 2,300 kW, cut-in wind speed 3.5 m/s, rated wind speed12.5 m/s, cut-out wind speed: 25 m/s. However^26^, has the power curve of the turbine SWT-2.3-113 (power 2,300 kW, diameter 113 m) of about same parameters, rated power 2,300 kW, cut-in wind speed 3 m/s, rated wind speed12.5 m/s and cut-out wind speed 25 m/s. As a first approximation, the SWT-2.3-113 power curve is used in lieu of the SWT-2.3-101 power curve.

**Figure 5:**
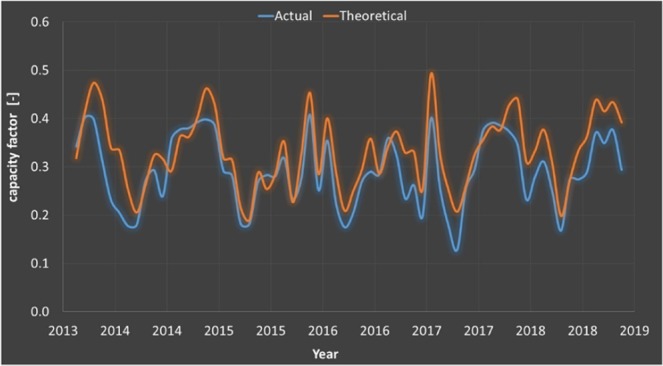
Los Vientos 1A measured and computed monthly capacity factors. Images reproduced modified after^38^.

Figure 5 presents a comparison of measured and computed electricity production for Los Vientos 1A during all the years of operation, and the measured and computed capacity factors. The images are reproduced modified after^38^. Further information may be found in^38^.

The theoretical values are expected to be larger than the actual values. Despite the method being quite simple, the wind resource is approximated similarly to the wind turbine curve, the details of terrain and turbine relative location are unknown, and the predicted values are usually slightly in excess than the measured values, with closely followed fluctuations in the electricity production. This validation supports the opportunity to assess the effect of the detailed wind turbine power curve on the capacity factor by using this model.

**Figure 6:**
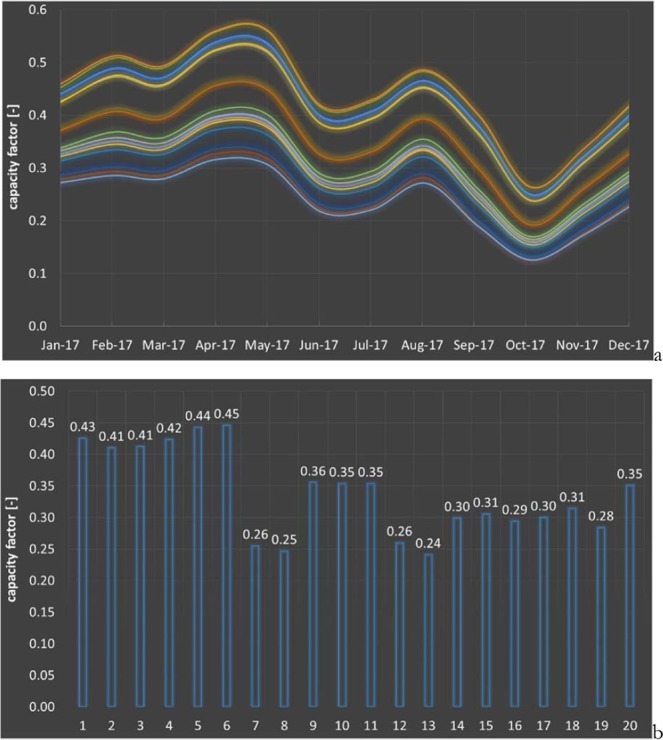
Monthly capacity factors of different turbines located in Los Vientos 1A over the year 2017 (**a**) and annual capacity factors (**b**).

Figure 6 presents the computed monthly capacity factors over the year 2017 for the turbines of Fig. 4 having a rotor axis placed at the same hub height of 100 m in the Los Vientos 1A location, plus the computed annual capacity factors for the same year. The maximum, minimum and average capacity factors for the hypothetical wind energy facilities built in the Los Vientos 1A location, with turbines 1–20, are 44.6%, 24.1%, and 33.9%. Thus, the difference between designs is quite considerable. Even turbines having about the same rated wind speed may indeed produce quite different capacity factors, as the actual shape of a turbine power curve having the same cut-in and rated speed still matters. Turbines have peak efficiency significantly variable between one design and the other. The net effect of a different turbine is to shift up and down the electricity production, without significantly affecting the shape of the curve.

The monthly capacity factors of the modeled 20 different turbines located in Los Vientos during 2017 have been 12.48% to 56.15%, with an average of 33.88%.

### The proposed new assessment of the cost and performance of wind energy facilities

Not only the forecasted CAPEX and annual average capacity factors of ^7^ are optimistic, but the production of electricity is also intermittent and unpredictable, and this must be accounted for. The variability is only minimally shown by the analysis of low frequency (monthly) data. A much higher frequency, 1 minute of less sampling frequency, is indeed needed to appreciate the variability. A procedure is thus proposed to correct cost and performance assessment including variability.

As wind energy facilities work not only with annual average capacity factors about 0.34, but also with high-frequency standard deviations of same order of magnitude, for about unity coefficients of variability^42^, with no certainty of any given production, at any time, the forecasted CAPEX cannot be compared with the CAPEX of, for example, of a combined cycle gas turbine power plant, of annual capacity factor in excess of 0.9, and high predictability of power output.

An energy storage allowance should be included to make meaningful the comparison between different energy sources, accounting for the variability.

While the actual costs are not examined in this manuscript, it has been shown as the annual average capacity factors are about 0.15 to 0.5, average 0.34, quite far from the forecasted values. Hence, the forecasted CAPEX should be also corrected for their actual annual average capacity factors, in addition to the allowance for energy storage.

It has been also shown, unfortunately only based on a low-frequency (monthly) statistic, as the capacity factors also vary from year to year and from month to month additional to facility-by-facility.

Different wind energy facilities, made up of different turbines of same nominal capacity, located at different hub height, in places of different wind energy resource, placed on different land sizes, more or less packed, and suffering of different topographies, all have the same nominal total capacity, but then they have much different actual production of electricity.

By looking at grid average values, for what concerns the cost, if CAPEX is the cost per unit nominal power, P_i_ is this nominal power, and ε_i_ is the annual capacity factor of the facility i, then the average CAPEX over all the n facilities connected to the same grid should be taken as:

4 $$CAPE{X}^{\ast }=\frac{{\sum }_{i=1}^{n}\frac{CAPE{X}_{i}}{{\varepsilon }_{i}}\cdot {\varepsilon }_{i}\cdot {P}_{i}}{{\sum }_{i=1}^{n}\,{\varepsilon }_{i}\cdot {P}_{i}}=\frac{{\sum }_{i=1}^{n}\,CAPE{X}_{i}\cdot {P}_{i}}{{\sum }_{i=1}^{n}\,{\varepsilon }_{i}\cdot {P}_{i}}$$

This number should be then corrected for the energy storage allowance.

Also looking at the grid average values for what concerns performance, at least two parameters should be considered. One parameter is the ratio of the annual average actual generating power to the nominal power, i.e. the annual average capacity factor, and one additional parameter is introduced to represent the variability about the annual average value. This may be the standard deviation of the capacity factor, computed by using a high-frequency statistic^42^. Both the annual average and the variability parameter of the capacity factor should be measured for every wind energy facility, preferably with high frequency, ideally every minute of even less. The standard deviation for every facility is

5 $${\rm{s}}=\sqrt{\frac{1}{m-1}\mathop{\sum }\limits_{i=1}^{m}{({\varepsilon }_{i}-\varepsilon )}^{2}}$$

where [ε_i_, i = 1, …., m] are the observed values at the sampling frequency (preferably every minute) over a year, and

6 $$\varepsilon =\frac{1}{m}\mathop{\sum }\limits_{i=1}^{m}\,{\varepsilon }_{i}$$

is the mean value of the observations, with *m* the number of observations. The capacity factors of the different wind energy facilities in the statistical sample are weighted on the electricity generated:

7 $${{\boldsymbol{\varepsilon }}}^{\ast }=\frac{{\sum }_{{\boldsymbol{i}}=1}^{{\boldsymbol{n}}}\,{{\boldsymbol{\varepsilon }}}_{{\boldsymbol{i}}}\cdot {{\boldsymbol{\varepsilon }}}_{{\boldsymbol{i}}}\cdot {{\boldsymbol{P}}}_{{\boldsymbol{i}}}}{{\sum }_{{\boldsymbol{i}}=1}^{{\boldsymbol{n}}}\,{{\boldsymbol{\varepsilon }}}_{{\boldsymbol{i}}}\cdot {{\boldsymbol{P}}}_{{\boldsymbol{i}}}}$$

Finally,

8 $${s}^{\ast }=\frac{{\sum }_{i=1}^{n}\,{s}_{i}\cdot {\varepsilon }_{i}\cdot {P}_{i}}{{\sum }_{i=1}^{n}\,{\varepsilon }_{i}\cdot {P}_{i}}$$

The only reasonable performance evaluation of a wind energy facility can be made comparing the annual average capacity factor and the standard deviation of the high-frequency distribution with the averaged values of all the facilities connected to the same grid.

## Discussion

In the case study of the 64 largest wind energy facilities in the continental US excluding Alaska, over the years 2013–2017, the annual capacity factors have been between 0.15 to 0.50, with an average of 0.34. The best performing wind energy facilities are aligned with the TRG values of ^7^, but the other wind energy facilities, the majority, are performing less.

In the 4 units of Los Vientos, TX, the annual capacity factors of 2017 vary from a maximum of 0.4858% to a minimum of 0.2084%, with an average of 0.3785%. In this TRG5 location of wind speed range 6.9–11.1 m/s, and weighted average wind speed 7.5 m/s, from^7^ the weighted average capacity factor is expected to be 0.407%. Also from these data, the capacity factors of ^7^ appear to be optimistic.

Similarly, optimistic is the CAPEX of ^7^, which is referred to the nominal rather than the actual generating capacity, with the first number much larger than the second, almost three times. Wind energy facilities are not nuclear power plants, that work on average at capacity factors about 0.92^43^, with small differences between one plant and the other, nor they are combined cycle gas turbines power plants, that also may work above 0.9 and are highly predictable. From Table 1, the capacity factors are 0.32 to 0.38 on average, depending on the year, and strongly variable between different wind farms, from 0.15 to 0.50.

The CaPEX of^7^ given as the cost per unit nominal power, should be replaced by the cost per unit actual power. This is simply accomplished by dividing the CAPEX of a facility by the capacity factor of that facility. But then, this CAPEX should also be corrected to include the grid average allowance for energy storage.

Theoretically, the annual capacity factors of 2017 of the 20 turbines considered, placed at the same hub height in Los Vientos vary from a maximum of 0.446 to a minimum of 0.241, with an average of 0.339. The reduced annual average capacity factor is likely partly because of the shape of the turbines’ power curve, plus the influence of hub height, with orography and relative position of the turbines, plus a variation of wind direction and air density, and turbulence, playing the rest.

The further uptake of wind energy calls for a better statistic, accounting for the actual cost referred to the actual annual average generating power, plus the actual capacity factor, and parameters describing the oscillations about the annual value of the capacity factor, computed by using the highest possible sampling frequency. Variability affects the actual cost of electricity. The standard deviation about the mean value is one simple parameter describing this variability.

The proposed statistic may permit to better assess the performance of the grid average wind energy facility and to individuate those who perform above, or below average, fostering cost reduction, as well as improving the ratio of actual annual average to nominal generating capacity, and reducing the fluctuations.

The case of the Australian National Electricity Market (NEM) grid, covering the most part of the population of the southeastern states of Australia, is the only example worldwide where data of power generation facilities, including wind and solar, is made available every 5 minutes by the Australian Energy Market Operator (AEMO). Ref. ^44^ collects the data of the AEMO in daily and monthly graphs. These data are presented as daily power graphs of 5-minute data, and monthly power graphs of 3-hour data, since 2018. Part of the data for 2018 is also available in CSV format. While the Australian data, is not as completed as the EIA US data, that is spanning the last 20 years, it may serve the purpose to show the effect of the sampling frequency in the wind energy time series.

The high-frequency data of the Australian NEM grid have been recently^45^ used to compute the growth factors for wind and solar installed capacity, and the storage actual power and energy needed, to make this specific grid wind and solar only. In the case of the US, now characterized by multiple grids, the exercise could be repeated once similar high-frequency data could be made available over one full year, for every grid, as well as for a hypothetical single grid. A single US grid could reduce the grid average variability of wind and solar, even if at the cost of larger transmission losses, and thus the energy storage requirements.

As shown in Ref. ^42^, the average capacity factors of wind are 0.3–0.38, and their standard deviations have about the same values, for coefficients of variations about unity. This indicates the extreme variability, that makes had to provide about constant outputs even over significant space average.

**Figure 7:**
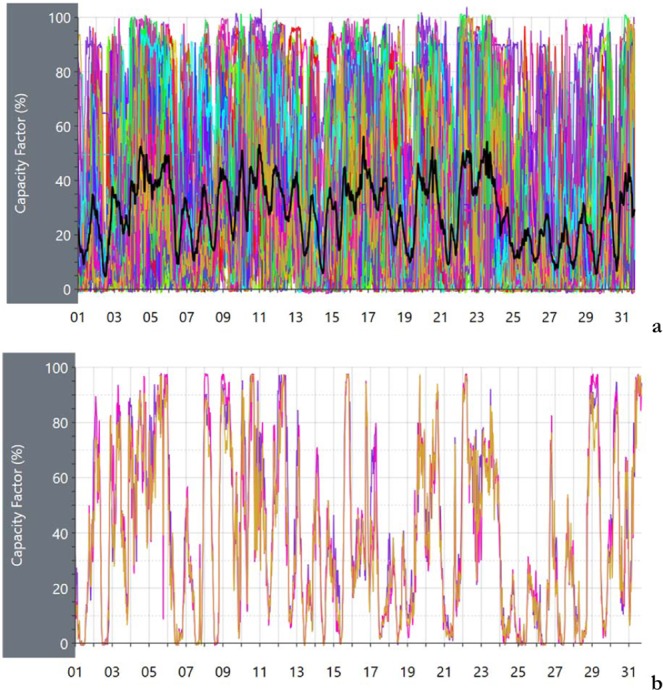
(**a**) Performance of the wind energy facilities connected to the electricity grid in south-east Australia over January 2020 (a mid-summer month). **(b)** Performance of the Hornsdale wind energy facility in South Australia over the same month. Images reproduced modified from^44^. anero.id/energy/. Credit Andrew Miskelly.

As an example, Fig. 7 presents the performance of all the wind energy facilities during January 2019, a midsummer month, as well as the performance of the 3 units of Hornsdale, in South Australia, one of the best performing wind farms of Australia, directly connected to the Hornsdale power reserve battery. The tick, black line is the grid-average. The grid average wind energy facility works often above 60%, but also below 5%, of the nominal power. This produces significant issues to the grid, and significant costs compensating for this variability.

Energy storage^46,47^ is thus essential for renewable energy. If we want all the energy of the grid wind and solar photovoltaics only, without any fully dispatchable combustion fuels power plants, then we do need massive energy storage^6^.

Renewable energy on demand from hydropower, which in Australia is presently 8%, but it is projected to reduce below 3% by 2030^48^, can help, but this is not enough. Additionally, to the hydro gravity facility being retrofitted with pumping capabilities to work as pumped hydro energy storage (PHES) facilities, other saltwater coastline PHES facilities, as well as battery facilities, are needed.

Presently^49^, battery energy storage in Australia is limited to about 200 MW power and about 200 MWh energy, also including the world’s largest battery, the 100 MW/129 MWh facility in South Australia.

Battery storage is indeed in its infantry. The batteries cannot be fully discharged, the world’s largest battery only lasts less than one hour and one half if discharged at a very optimistic peak power of 100 MW.

As shown in^45^, wind and solar capacities must be increased to 53.2 and 90.5 GW (growth factor 7.94) to fully cover the present NEM grid demand with additionally a minimum actual storage power of more than 45 GW net, and actual storable energy in excess of 3,500 GW·h. The nominal power and energy of the storage are much larger as it depends on the specific technology adopted, as for example battery storage only works at a fraction of the nominal power during charge and discharge, and only a fraction of the nominal capacity can be used, with round-trip efficiencies everything but unity. Worth to note, is also the length of the storage. As both wind and solar are strongly affected by seasonality, and the total production of wind and solar vary with the season, a wind and solar only grid also necessitate long term storage, that is an additional challenge not considered before.

When high-frequency data will be eventually made available for the US over a full year for all the wind and solar facilities connected to the same grid of given demand, then it will be possible to compute growth factors for wind and solar capacity, total power, and energy of the storage, cost of the storage, and finally attribute this cost to every facility, inversely proportional to the annual mean capacity factor, and directly proportional to the standard deviation about this value.

## Conclusions

The CAPEX and annual average capacity factors forecasted in technology assessments for wind are optimistic. The production of electricity from wind energy facilities is intermittent and unpredictable, and this must be accounted for, the same as the actual annual average generating power.

The CAPEX of a wind energy facilities that are working not only with annual average capacity factors about 0.34, but also with high-frequency standard deviations of same order of magnitude of the mean capacity factors, for about unity coefficients of variability, and no certainty of any given production at any time, cannot be compared with the CAPEX of power plants of annual capacity factor in excess of 0.9 and high predictability. An energy storage allowance should be included to make the comparison meaningful, accounting for the variability.

While the actual costs are not examined, it is also shown as the annual average capacity factors are about 0.15 to 0.5, average 0.34, quite far from the forecasted values. It is also shown based on a low-frequency (monthly) statistic as the capacity factors also vary from year-by-year and from month-by-month.

Different wind energy facilities, of different turbines albeit of same nominal capacity, located at different hub height, in places of different wind energy resource, and sited on different terrains, more or less packed together, may all have the same nominal capacity, but then they have much different actual production of electricity. To take the sum of the nominal power of all the turbines as the reference power of a wind energy facility is an oversimplification not recognizing the value of better design, thus not rewarding the developers that are building better wind energy facilities.

The energy storage issue should be acknowledged the sooner the better, as without, wind and solar energy are unable to supply the energy needed by a balanced grid without combustion fuels.

The novelty of the present work is the recognition of the variability of wind power generation as a performance and cost parameter, and the proposal of a practical way to progress the design of the storage and its cost attribution to the generating facilities.
